# A Novel Effective Approach for Solving Fractional Nonlinear PDEs

**DOI:** 10.1155/2014/647492

**Published:** 2014-10-29

**Authors:** Hossein Aminikhah, Nasrin Malekzadeh, Hadi Rezazadeh

**Affiliations:** Department of Applied Mathematics, School of Mathematical Sciences, University of Guilan, P.O. Box 1914, Rasht 41938, Iran

## Abstract

The present work introduces an effective modification of homotopy perturbation method for the solution of nonlinear time-fractional biological population model and a system of three nonlinear time-fractional partial differential equations. In this approach, the solution is considered a series expansion that converges to the nonlinear problem. The new approximate analytical procedure depends only on two iteratives. The analytical approximations to the solution are reliable and confirm the ability of the new homotopy perturbation method as an easy device for computing the solution of nonlinear equations.

## 1. Introduction

In recent years, fractional differential equations have played an important role in different research areas such as mechanics, electricity, biology, economics, notably control theory, and signal and image processing [1–5]. Many phenomena can be described very successfully by models using mathematical tools from fractional calculus, that is, the theory of derivatives and integrals of fractional order. The most important advantage of using fractional differential equations in these and other applications is their nonlocal property. It is well known that the integer order differential operator is a local operator, but the fractional-order differential operator is nonlocal. This means that the next state of a system depends not only upon its current state but also upon all of its historical states. In fact, this is the main reason why differential operators of fractional order provide an excellent instrument for description of memory and hereditary properties of various mathematical, physical, and engineering processes. The reader is asked to refer to [6–9] in order to know more details about the fractional DEs, including their history and kinds, their existence and uniqueness of solutions, and their applications and methods of solutions.

During the last few years, the numerical methods and exact solution methods have been proposed to solve fractional differential equations, for example, the Adomian decomposition method [10], the homotopy perturbation method [11, 12], the variational iteration method [13, 14], the differential transform method [15, 16], the *G*′/*G* method [17, 18], the first integral method [19], and the exp-function method [20]. Aminikhah and Hemmatnezhad [21] and Aminikhah and Biazar [22] proposed a new homotopy perturbation method to obtain the exact and numerical solutions of ordinary differential equation.

In this paper, we will implement the new homotopy perturbation method to obtain the approximate solution for the following time-fractional derivative nonlinear partial differential equation.

(i) The fractional-order biological population equation as a nonlinear model is as follows [23, 24]:
  (1) Dtαu=(u2)xx+(u2)yy+f(u), t>0,iiiiiiiiiiiiiiiiiiiiiiiiiiiiix,y∈R, 0<α≤1f(u)=hua(1−rub),u(x,y,0)=g(x,y),

where *u* denotes population density, *f* represents the population supply due to births and deaths, *h*, *a*, *r*, and *b* are real numbers, *g* is given initial condition, and *D*_*t*_^*α*^ denotes the differential operator in the sense of Caputo.

(ii) The system of three nonlinear time-fractional partial equations which have been widely discussed in the literature [25, 26] is as follows:
  (2) Dtαu=−vxwy+vywx−u,Dtαv=−wxuy−wyux+v,Dtαw=−uxvy−uyvx+w,iiiiiiiiiiiiiit>0,  0<α≤1,

with initial conditions

(3) $$\begin{array}{c} u\left(x,y,0\right)=h_{1}\left(x,y\right),\\ v\left(x,y,0\right)=h_{2}\left(x,y\right),\\ w\left(x,y,0\right)=h_{3}\left(x,y\right), \end{array}$$

where *D*_*t*_^*α*^ denotes the differential operator in the sense of Caputo.

The rest of this paper is organized as follows. In Section 2, the properties of fractional derivative are described. In Section 3, basic ideas of new homotopy perturbation method for solving fractional partial differential equations are presented. In Section 4, the application of new homotopy perturbation method for solving the nonlinear fractional biological population equation and the system of three nonlinear time-fractional partial equations are presented. Finally, we give a brief conclusion in the last section.

## 2. Background on Fractional Derivatives

The history of fractional calculus is more than three centuries old, yet only in the past 20 years has the field received much attention and interest. The reader may refer to [8, 9]. There are several definitions for fractional differential equations. These definitions include Grunwald-Letnikov, Riemann-Liouville, Caputo, Weyl, Marchaud, and Riesz fractional derivatives; among those Riemann-Liouville and Caputo fractional derivatives are the most popular. The differential equations defined in terms of Riemann-Liouville derivatives require fractional initial conditions whereas the differential equations defined in terms of Caputo derivatives require boundary conditions involving integer order derivatives, which have clear physical interpretations. For this reason, Caputo fractional derivatives are popular among scientists and engineers.

Now, we give some basic definitions and properties of the fractional calculus theory used in this work.

**Definition 1 .**

The Riemann-Liouville fractional integral operator *J* ^*μ*^ of order *μ* on the usual Lebesgue space *L*_1_[*a*, *b*] is given by

(4) $$\begin{array}{c} J^{\mu }f\left(x\right)=\frac{1}{\Gamma \left(\mu \right)}\overset{x}{\underset{0}{\int }}{\left(x-t\right)}^{\mu -1}f\left(t\right)dt,\;\mu >0,\\ J^{0}f\left(x\right)=f\left(x\right). \end{array}$$

It has the following properties:

(i) *J* ^*μ*^ exists for any *x* ∈ [*a*, *b*],
(ii) *J* ^*μ*^ *J* ^*β*^ = *J* ^*μ*+*β*^,
(iii) *J* ^*μ*^ *J* ^*β*^ = *J* ^*β*^ *J* ^*μ*^,
(iv) *J* ^*μ*^ *J* ^*β*^ *f*(*x*) = *J* ^*β*^ *J* ^*μ*^ *f*(*x*),
(v) *J* ^*μ*^(*x* − *a*)^*γ*^ = (Γ(*γ* + 1)/Γ(*μ* + *γ* + 1))(*x* − *a*)^*μ*+*γ*^,

where *f* ∈ *L*_1_[*a*, *b*], *μ*, *β* ≥ 0, and *γ* > −1.

**Definition 2 .**

The Caputo definition of fractal derivative operator is given by

(5) Dμf(x)=Jm−μDnf(x)=1Γ(m−μ)∫0t(x−τ)m−μ−1fm(τ)dτ,

where *m* − 1 < *μ* ≤ *m*,  *m* ∈ *N*, *x* > 0, and *f* ∈ *L*_1_[*a*, *b*].

**Remark 3 .**

For *n* to be the smallest integer that exceeds *α*, the Caputo time-fractional derivative operator of *α* > 0 is defined as

(6) Dtαu(x,y,t)=∂αu(x,y,t)∂tα={1Γ(n−α)∫0t(t−τ)n−α−1∂nu(x,y,τ)∂tndτ,n−1<α<n∂nu(x,y,t)∂tn,α=n∈N.

## 3. Analysis of New Homotopy Perturbation Method

In this section, we construct the solution of system of partial differential equations with time-fractional derivative by extending the idea of [21, 22]. Let us consider the system of nonlinear fractional differential equations:

(7) $$\begin{array}{c} D_{t}^{\alpha }u_{i}\left(x,t\right)=A_{i}\left(u_{i}\right)+f_{i}\left(t,x\right),\;x,t\in \Omega ,\;\;i=1,2,\ldots ,n, \end{array}$$

with the following initial conditions:

(8) $$\begin{array}{c} u_{i}\left(x,0\right)=\alpha_{i},\;i=1,2,\ldots ,n, \end{array}$$

where *A*_*i*_ are the operators, *f*_*i*_ are known functions, and *u*_*i*_ are sought functions. Assume that operators *A*_*i*_ can be written as *A*_*i*_(*u*_*i*_) = *L*_*i*_(*u*_*i*_) + *N*_*i*_(*u*_*i*_), where *L*_*i*_ are the linear operators and *N*_*i*_ are the nonlinear operators. Hence, (7) can be rewritten as follows:

(9) $$\begin{array}{c} D_{t}^{\alpha }u_{i}\left(x,t\right)=L_{i}\left(u_{i}\right)+N_{i}\left(u_{i}\right)+f_{i}\left(x,t\right). \end{array}$$

For solving system (7) by NHPM [22] we construct the following homotopy:

(10) H(Ui;p)=(1−p)(DtαUi(x,t)−ui,0) +p(Dtαui(x,t)−Li(Ui)−Ni(Ui)−fi(t,x))=0,

where *p* ∈ [0,1]  is an embedding or homotopy parameter, *H*(*t*, **x**; *p*) : *Ω* × [0,1] → *R*, and *u*_*i*,0_ are the initial approximation of solution of the problem in (9).

Clearly, the homotopy equations *H*(*U*_*i*_; 0) = 0 and *H*_*i*_(*U*_*i*_; 1) = 0 are equivalent to the equations *D*_*t*_^*α*^ *U*_*i*_(**x**, *t*) − *u*_*i*,0_ = 0 and *D*_*t*_^*α*^ *U*_*i*_(**x**, *t*) − *L*_*i*_(*U*_*i*_) + *N*_*i*_(*U*_*i*_) + *f*_*i*_(*t*, **x**) = 0, respectively. Thus, a monotonous change of parameter *p* from zero to one corresponds to a continuous change of the trivial problem *D*_*t*_^*α*^ *U*_*i*_(**x**, *t*) − *u*_*i*,0_ = 0 to the original problem. Next, we assume that the solution of equation *H*(*U*_*i*_, *p*) can be written as a power series in embedding parameter *p*, as follows:

(11) $$\begin{array}{c} U_{i}=U_{i,0}+pU_{i,1},\;i=1,2,\ldots ,n. \end{array}$$

Now, let us write (11) in the following form:

(12) $$\begin{array}{c} D_{t}^{\alpha }U_{i}\left(x,t\right)=u_{i,0}+p\left(L_{i}\left(U_{i}\right)+N_{i}\left(U_{i}\right)+f_{i}\left(t,x\right)\right). \end{array}$$

Applying the inverse operator *J*_*t*_^*α*^ which is the Riemann-Liouville fractional integral of order *α* ≥ 0, on both sides of (12), we have

(13) Ui(x,t)=Ui(x,0)+Jtαui,0 +pJtα(Li(Ui)+Ni(Ui)+fi(t,x)).

Suppose that the initial approximation of (9) has the form

(14) $$\begin{array}{c} u_{i,0}\left(x,t\right)=\overset{\infty }{\underset{n=0}{\sum }}a_{i,n}\left(x\right)p_{n}\left(t\right),\;i=1,2,\ldots ,n, \end{array}$$

where *a*_*i*,*n*_(**x**), *n* = 0,1, 2,…, are unknown coefficients and *p*_*n*_(*t*), *n* = 0,1, 2,…, are specific functions on the problem. By substituting (11) and (14) into (13), we get

(15) Ui,0+pUi,1 =Ui(x,0)+Jtα(∑n=0∞ai,n(x)pn(t))  +pJtα(Li(Ui,0+pUi,1)+Ni(Ui,0+pUi,1)+fi(t,x)).

Equating the coefficients of like powers of *p*, we get the following set of equations:

(16) coefficient  of  p0: Ui,0(x,t)=Ui(x,0)+Jtα(∑n=0∞ai,n(x)pn(t)),coefficient  of  p1: Ui,1(x,t)=Jtα(Li(Ui,0)+Ni(Ui,0)+fi(t,x)).

Now, we solve these equations in such a way that *U*_*i*,1_(**x**, *t*) = 0. Therefore, the approximate solution may be obtained as

(17) $$\begin{array}{c} u_{i}\left(x,t\right)=U_{i,0}\left(x,t\right)=U_{i}\left(x,0\right)+J_{t}^{\alpha }\left(\overset{\infty }{\underset{n=0}{\sum }}a_{i,n}\left(x\right)p_{n}\left(t\right)\right). \end{array}$$

## 4. Applications

In this section, we present some examples with analytical solution to show the efficiency of methods described in the previous section for solving (1) and (2).

**Example 1 .**

Let us consider (1) with *a* = 1, *r* = 0, and $g(x,y)=\sqrt{xy}$, then we have

(18) Dtαu(x,y,t)=∂2(u2(x,y,t))∂x2+∂2(u2(x,y,t))∂y2 +hu(x,y,t),u(x,y,0)=xy.

The exact solution of (18), for the special case *α* = 1, is $u(x,y,t)=\sqrt{xy}e^{ht}$.

To obtain the solution of (18) by new homotopy perturbation method, we construct the following homotopy:

(19) (1−p)(DtαU(x,y,t)−u0(x,y,t)) +p(DtαU(x,y,t)−∂2(U2(x,y,t))∂x2    −∂2(U2(x,y,t))∂y2−hU(x,y,t))=0.

Applying the inverse operator *J*_*t*_^*α*^ of *D*_*t*_^*α*^ on both sides of (19), we obtain

(20) U(x,y,t)=U(x,y,0)+Jtαu0(x,y,t) −pJtα(u0(x,y,t)−∂2(U2(x,y,t))∂x2iiiiiiiiiiiiiiiii−∂2(U2(x,y,t))∂y2−hU(x,y,t)).

The solution of (21) has the following form:

(21) $$\begin{array}{c} U\left(x,y,t\right)=U_{0}\left(x,y,t\right)+pU_{1}\left(x,y,t\right). \end{array}$$

Substituting (21) into (20) and equating the coefficients of like powers of *p*, we get the following set of equations:

(22) U0(x,y,t)=U(x,y,0)+Jtαu0(x,y,t),U1(x,y,t)=Jtα(−u0(x,y,t)+∂2(U2(x,y,t))∂x2   +∂2(U2(x,y,t))∂y2+hU(x,y,t)).

Assuming *u*_0_(*x*, *y*, *t*) = ∑_*n*=0_^*∞*^ *a*_*n*_(*x*, *y*)*p*_*n*_(*t*), *p*_*n*_(*t*) = *t* ^*nα*^, and *U*(*x*, *y*, 0) = *u*(*x*, *y*, 0) and solving (22) for *U*_1_(*x*, *y*, *t*) lead to the following result:

(23) U1(x,y,t)=(−a0(x,y)+hxy)tαΓ(α+1) +(2(∂a0(x,y)/∂y)xxy−12a0(x,y)y2(xy)3/2   +2(∂a0(x,y)/∂x)yxy+ha0(x,y)+2xy∂2a0(x,y)∂y2   −a1(x,y)−12a0(x,y)x2(xy)3/2   + 2xy∂2a0(x,y)∂x2)Γ(α+1)t2αΓ(2α+1)   +((∂a1(x,y)/∂y)xxy−14a1(x,y)y2(xy)3/214a1(x,y)x2(xy)3/2iiiiiiiiiiiiiii+2(∂a0(x,y)∂y)2+2a0(x,y)∂2a0(x,y)∂y2iiiiiiiiiiiiiii+xy∂2a1(x,y)∂y2+2(∂a0(x,y)∂x)2iiiiiiiiiiiiiii+12ha1(x,y)+2a0(x,y)∂2a0(x,y)∂x2iiiiiiiiiiiiiii+(∂a1(x,y)/∂x)yxy+xy∂2a1(x,y)∂x2iiiiiiiiiiiiiii−a2(x,y)−14a1(x,y)x2(xy)3/2)Γ(2α+1)t3αΓ(3α+1)   +(a0(x,y)∂2a1(x,y)∂y2iiiiiiiiiiiiiiii+ 23xy∂2a2(x,y)∂y2⋯−  a3(x,y))   ×Γ(3α+1)t4αΓ(4α+1)+⋯.

Vanishing *U*_1_(*x*, *y*, *t*) lets the coefficients *a*_*i*_, *i* = 0,1, 2,…, have the following values:

(24) $$\begin{array}{c} a_{0}\left(x,y\right)=h\sqrt{xy},\;\;a_{1}\left(x,y\right)=h^{2}\sqrt{xy},\\ a_{2}\left(x,y\right)=\frac{1}{2!}h^{3}\sqrt{xy},\;\;a_{3}\left(x,y\right)=\frac{1}{3!}h^{4}\sqrt{xy},\\ a_{4}\left(x,y\right)=\frac{1}{4!}h^{5}\sqrt{xy},\ldots . \end{array}$$

Therefore, we obtain the solutions of (18) as

(25) u(x,y,t)=xy+hxytαΓ(α+1) +h2xyΓ(α+1)t2αΓ(2α+1)+12!h3xyΓ(2α+1)t3αΓ(3α+1) +13!h4xyΓ(3α+1)t4αΓ(4α+1)+⋯=xy(1+∑n=1∞Γ((n−1)α+1)(htα)n(n−1)!Γ(nα+1)).

If *α* = 1, we have

(26) u(x,y,t)=xy(1+ht+h2t22!+h3t33!+⋯)=xyeht,

which is an exact solution.

**Example 2 .**

Consider (1) with *a* = 1, *b* = 1, and $g(x,y)=e^{\sqrt{hr/8}(x+y)}$, then we have

(27) Dtαu(x,y,t)=∂2(u2(x,y,t))∂x2+∂2(u2(x,y,t))∂y2 +hu(x,y,t)(1−ru(x,y,t));

subject to the initial condition

(28) $$\begin{array}{c} u\left(x,y,0\right)=e^{\sqrt{hr/8}(x+y)}. \end{array}$$

The exact solution of (27), for the special case *α* = 1, is

(29) $$\begin{array}{c} u\left(x,y,t\right)=e^{\sqrt{hr/8}(x+y)+ht}. \end{array}$$

To obtain the solution of (27) by the new homotopy perturbation method, we construct the following homotopy:

(30) (1−p)(DtαU(x,y,t)−u0(x,y,t))  +p(DtαU(x,y,t)∂2(U2(x,y,t))∂x2iiiiiiiiiiiiiiiiii−∂2(U2(x,y,t))∂x2−∂2(U2(x,y,t))∂y2iiiiiiiiiiiiiiiiii−∂2(U2(x,y,t))∂x2hU(x,y,t)(1−rU(x,y,t)))=0.

Applying the inverse operator *J*_*t*_^*α*^ of *D*_*t*_^*α*^ on both sides of (30), we obtain

(31) U(x,y,t)=U(x,y,0)+Jtαu0(x,y,t) −pJtα(u0(x,y,t)∂2(U2(x,y,t))∂x2iiiiiiiiiiiiiiiii−∂2(U2(x,y,t))∂x2−∂2(U2(x,y,t))∂y2iiiiiiiiiiiiiiiii∂2(U2(x,y,t))∂x2−hU(x,y,t)(1−rU(x,y,t))).

The solution of (31) has the following form:

(32) $$\begin{array}{c} U\left(x,y,t\right)=U_{0}\left(x,y,t\right)+pU_{1}\left(x,y,t\right). \end{array}$$

Substituting (32) into (33) and equating the coefficients of like powers of *p*, we get the following set of equations:

(33) U0(x,y,t)=U(x,y,0)+Jtαu0(x,y,t),U1(x,y,t)=Jtα(−u0(x,y,t)+∂2(U2(x,y,t))∂x2iiiiiiiiiiii+∂2(U2(x,y,t))∂y2iiiiiiiiiiii∂2(U2(x,y,t))∂y2+ hU(x,y,t)(1−rU(x,y,t))).

Assuming *u*_0_(*x*, *y*, *t*) = ∑_*n*=0_^10^ *a*_*n*_(*x*, *y*)*p*_*n*_(*t*), *p*_*n*_(*t*) = *t* ^*nα*^, *U*(*x*, *y*, 0) = *u*(*x*, *y*, 0), and solving (33) for *U*_1_(*x*, *y*, *t*) lead to the following result:

(34) U1(x,y,t)=(−a0(x,y)+(ehr/8(x+y))2hr  + h(ehr/8(x+y)−r(ehr/8(x+y))2))tαΓ(α+1) +(2ehr/8(x+y)∂2a0(x,y)∂x2+2hrehr/8(x+y)∂a0(x,y)∂x   +h(−2ehr/8(x+y)ra0(x,y)+a0(x,y))   +2hrehr/8(x+y)∂a0(x,y)∂y   +12hrehr/8(x+y)a0(x,y)   − a1(x,y)+2ehr/8(x+y)∂2a0(x,y)∂y2)Γ(α+1)t2αΓ(2α+1) +(ehr/8(x+y)∂2a1(x,y)∂x2−a2(x,y)+2(∂a0(x,y)∂y)2   +h(−ehr/8(x+y)ra1(x,y)−(a0(x,y))2r+12iiiiiiiiiiiiiiiiiiiii−(a0(x,y))2r+12a1(x,y))   +14hrehr/8(x+y)a1(x,y)+2a0(x,y)∂2a0(x,y)∂y2   +ehr/8(x+y)∂2a1(x,y)∂y2+2(∂a0(x,y)∂x)2   +2a0(x,y)∂2a0(x,y)∂x2   +122hrehr/8(x+y)∂a1(x,y)∂y   + 122hrehr/8(x+y)∂a1(x,y)∂x)Γ(2α+1)t3αΓ(3α+1) +(132hrehr/8(x+y)∂a2(x,y)∂y−a3(x,y)   +···+23ehr/8(x+y)∂2a2(x,y)∂y2)Γ(3α+1)t4αΓ(4α+1)+⋯.

Vanishing *U*_1_(*x*, *y*, *t*) lets the coefficients *a*_*i*_, *i* = 0,1, 2,…, have the following values:

(35) $$\begin{array}{c} a_{0}\left(x,y\right)=he^{\sqrt{hr/8}(x+y)},\;\;a_{1}\left(x,y\right)=h^{2}e^{\sqrt{hr/8}(x+y)},\\ a_{2}\left(x,y\right)=\frac{1}{2!}h^{3}e^{\sqrt{hr/8}(x+y)},\;\;a_{3}\left(x,y\right)=\frac{1}{3!}h^{4}e^{\sqrt{hr/8}(x+y)},\\ a_{4}\left(x,y\right)=\frac{1}{4!}h^{5}e^{\sqrt{hr/8}(x+y)},\ldots . \end{array}$$

Therefore, we obtain the solution of (27) as

(36) u(x,y,t)=ehr/8(x+y)+hehr/8(x+y)tαΓ(α+1) +h2ehr/8(x+y)Γ(α+1)t2αΓ(2α+1) +12!h3ehr/8(x+y)Γ(2α+1)t3αΓ(3α+1) +13!h4ehr/8(x+y)Γ(3α+1)t4αΓ(4α+1)−···=ehr/8(x+y)(1+∑n=1∞Γ((n−1)α+1)(htα)n(n−1)!Γ(nα+1)).

If we put *α* → 1 in (36) or solve (30) with *α* = 1, we obtain the exact solution

(37) u(x,y,t)=ehr/8(x+y)(1+ht+h2t22!+h3t33!+⋯)=ehr/8(x+y)+ht.

**Example 3 .**

Let us consider (2) with *h*_1_(*x*, *y*) = *e* ^(*x*+*y*)^, *h*_2_(*x*, *y*) = *e* ^(*x*−*y*)^, and *h*_3_(*x*, *y*) = *e* ^(−*x*+*y*)^. Therefore, we have

(38) Dtαu(x,y,t)=−∂v(x,y,t)∂x∂w(x,y,t)∂y +∂v(x,y,t)∂y∂w(x,y,t)∂x−u(x,y,t),Dtαv(x,y,t)=−∂w(x,y,t)∂x∂u(x,y,t)∂y −∂w(x,y,t)∂y∂u(x,y,t)∂x+v(x,y,t),Dtαw(x,y,t)=−∂u(x,y,t)∂x∂v(x,y,t)∂y −∂u(x,y,t)∂y∂v(x,y,t)∂x+w(x,y,t)

subject to the initial conditions

(39) $$\begin{array}{c} u\left(x,y,0\right)=e^{x+y},\\ v\left(x,y,0\right)=e^{x-y},\\ w\left(x,y,0\right)=e^{-x+y}. \end{array}$$

The exact solution of (38), for the special case *α* = 1, is

(40) $$\begin{array}{c} u\left(x,y,t\right)=e^{x+y-t},\\ v\left(x,y,t\right)=e^{x-y+t},\\ w\left(x,y,t\right)=e^{-x+y+t}. \end{array}$$

To obtain the solution of (38) by new homotopy perturbation method, we construct the following homotopy:

(41) (1−p)(DtαU(x,y,t)−u0(x,y,t)) +p(DtαU(x,y,t)+∂V(x,y,t)∂x∂W(x,y,t)∂yiiiiiiiiiiiii− ∂V(x,y,t)∂y∂W(x,y,t)∂x+U(x,y,t))=0,(1−p)(DtαV(x,y,t)−v0(x,y,t)) +p(DtαV(x,y,t)+∂W(x,y,t)∂x∂U(x,y,t)∂yiiiiiiiiiiiiiii+  ∂W(x,y,t)∂y∂U(x,y,t)∂x−V(x,y,t))=0,(1−p)(DtαW(x,y,t)−w0(x,y,t)) +p(DtαW(x,y,t)+∂U(x,y,t)∂x∂V(x,y,t)∂yiiiiiiiiiiiiii+ ∂U(x,y,t)∂y∂V(x,y,t)∂x−W(x,y,t))=0.

Applying the inverse operator *J*_*t*_^*α*^ of *D*_*t*_^*α*^ on both sides of the above equation, we obtain

(42) U(x,y,t) =U(x,y,0)+Jtαu0(x,y,t)  −pJtα(u0(x,y,t)+∂V(x,y,t)∂x∂W(x,y,t)∂yiiiiiiiiiiiiiiiiiii+∂V(x,y,t)∂x∂W(x,y,t)∂y−∂V(x,y,t)∂y∂W(x,y,t)∂x+U(x,y,t)),V(x,y,t) =V(x,y,0)+Jtαv0(x,y,t)  −pJtα(v0(x,y,t)+∂W(x,y,t)∂x∂U(x,y,t)∂yiiiiiiiiiiiiiiiiiiiii+∂W(x,y,t)∂y∂U(x,y,t)∂x−V(x,y,t)),W(x,y,t) =W(x,y,0)+Jtαw0(x,y,t)  −pJtα(w0(x,y,t)+∂U(x,y,t)∂x∂V(x,y,t)∂yiiiiiiiiiiiiiiiiiiiii+∂U(x,y,t)∂y∂V(x,y,t)∂x−W(x,y,t)).

The solution of (42) has the following form:

(43) $$\begin{array}{c} U\left(x,y,t\right)=U_{0}\left(x,y,t\right)+pU_{1}\left(x,y,t\right),\\ V\left(x,y,t\right)=V_{0}\left(x,y,t\right)+pV_{1}\left(x,y,t\right),\\ W\left(x,y,t\right)=W_{0}\left(x,y,t\right)+pW_{1}\left(x,y,t\right). \end{array}$$

Substituting (43) into (42) and equating the coefficients of like powers of *p*, we get the following set of equations:

(44) U0(x,y,t)=U(x,y,0)+Jtαu0(x,y,t),V0(x,y,t)=V(x,y,0)+Jtαv0(x,y,t),W0(x,y,t)=W(x,y,0)+Jtαw0(x,y,t),U1(x,y,t) =Jtα(−u0(x,y,t)−∂V(x,y,t)∂x∂W(x,y,t)∂yiiiiiiiiiiiiiiiii+∂V(x,y,t)∂y∂W(x,y,t)∂x−U(x,y,t)),V1(x,y,t) =Jtα(−v0(x,y,t)−∂W(x,y,t)∂x∂U(x,y,t)∂yiiiiiiiiiiiiiiiii−∂W(x,y,t)∂y∂U(x,y,t)∂x+V(x,y,t)),W1(x,y,t) =Jtα(−w0(x,y,t)−∂U(x,y,t)∂x∂V(x,y,t)∂yiiiiiiiiiiiiiiiii∂U(x,y,t)∂x−∂U(x,y,t)∂y∂V(x,y,t)∂x+W(x,y,t)).

Assuming

(45) $$\begin{array}{c} u_{0}\left(x,y,t\right)=\overset{\infty }{\underset{n=0}{\sum }}a_{n}\left(x,y\right)p_{n}\left(t\right),\\ v_{0}\left(x,y,t\right)=\overset{\infty }{\underset{n=0}{\sum }}b_{n}\left(x,y\right)p_{n}\left(t\right),\\ w_{0}\left(x,y,t\right)=\overset{\infty }{\underset{n=0}{\sum }}c_{n}\left(x,y\right)p_{n}\left(t\right),\;\;p_{n}\left(t\right)=t^{n\alpha },\\ U\left(x,y,0\right)=u\left(x,y,0\right),\;\;V\left(x,y,0\right)=v\left(x,y,0\right),\\ W\left(x,y,0\right)=w\left(x,y,0\right) \end{array}$$

and solving the above equation for *U*_1_(*x*, *y*, *t*) and *V*_1_(*x*, *y*, *t*) and *W*_1_(*x*, *y*, *t*) lead to the following result:

(46) U1(x,y,t) =(−a0(x,y)−ex+y)tαΓ(α+1)  +(−ex−y∂c0(x,y)∂y−∂b0(x,y)∂xe−x+y−a1(x,y)iiiiiiIiiiiiii−a0(x,y)−∂b0(x,y)∂ye−x+yiiiiiiiiiiiiiii−ex−y∂c0(x,y)∂x)Γ(α+1)t2αΓ(2α+1)  +(−12ex−y∂c1(x,y)∂y−12∂b1(x,y)∂ye−x+yiiiiiiiiiiiiiii−12ex−y∂c1(x,y)∂x−12∂b1(x,y)∂xe−x+yiiiiiiiiiiiiiii−12a1(x,y)−∂b0(x,y)∂x∂c0(x,y)∂yiiiiiiiiiiiiiii+∂b0(x,y)∂y∂c0(x,y)∂x−a2(x,y))Γ(2α+1)t3αΓ(3α+1)  +(−13∂b2(x,y)∂xe−x+y+12∂b0(x,y)∂yiiiiiiiiiiiiiii×∂c1(x,y)∂x+⋯−a3(x,y))Γ(3α+1)t4αΓ(4α+1)+⋯,V1(x,y,t) =(−b0(x,y)+ex−y)tαΓ(α+1)  +(−e−x+y∂a0(x,y)∂x−∂c0(x,y)∂yex+yiiiiiiiiiiiiiii−∂c0(x,y)∂xex+y+b0(x,y)−b1(x,y)iiiiiiiiiiiiiii+∂a0(x,y)∂ye−x+y)Γ(α+1)t2αΓ(2α+1)  +(12e−x+y∂a1(x,y)∂y−12∂a1(x,y)∂xe−x+yiiiiiiiiiiiiiii−12ex+y∂c1(x,y)∂x−12∂c1(x,y)∂yex+yiiiiiiiiiiiiiii−12b1(x,y)−∂c0(x,y)∂x∂a0(x,y)∂yiiiiiiiiiiiiiii−∂c0(x,y)∂y∂a0(x,y)∂x−b2(x,y))Γ(2α+1)t3αΓ(3α+1)  +(−13∂a2(x,y)∂xe−x+y−12∂c1(x,y)∂x∂a0(x,y)∂yiiiiiiiiiiiiiii12∂c1(x,y)∂x+⋯−b3(x,y))Γ(3α+1)t4αΓ(4α+1)+⋯,W1(x,y,t) =(−c0(x,y)+e−x+y)tαΓ(α+1)  +(ex−y∂a0(x,y)∂x−∂a0(x,y)∂yex−yiiiiiiiiiiiiiii−∂b0(x,y)∂xex+y+c0(x,y)−c1(x,y)iiiiiiiiiiiiiii−∂b0(x,y)∂yex+y)Γ(α+1)t2αΓ(2α+1)  +(12ex−y∂a1(x,y)∂x−12∂b1(x,y)∂xex+yiiiiiiiiiiiiiii−12ex+y∂b1(x,y)∂y−12∂a1(x,y)∂yex−yiiiiiiiiiiiiiii+12c1(x,y)−∂a0(x,y)∂x∂b0(x,y)∂yiiiiiiiiiiiiiii−∂a0(x,y)∂y∂b0(x,y)∂x−c2(x,y))Γ(2α+1)t3αΓ(3α+1)  +(−13∂a2(x,y)∂xex−y−12∂b1(x,y)∂x∂a0(x,y)∂yiiiiiiiiiiiiiii−13∂a2(x,y)∂x+⋯−c3(x,y))Γ(3α+1)t4αΓ(4α+1)+⋯.

Vanishing *U*_1_(*x*, *y*, *t*), *V*_1_(*x*, *y*, *t*), and *W*_1_(*x*, *y*, *t*) lets the coefficients *a*_*i*_, *b*_*i*_, *c*_*i*_, *i* = 0,1, 2,…, have the following values:

(47) a0(x,y)=−ex+y,  a1(x,y)=ex+y,a2(x,y)=−12!ex+y,  a3(x,y)=13!ex+y,a4(x,y)=−14!ex+y,…,b0(x,y)=ex−y,  b1(x,y)=ex−y,b2(x,y)=12!ex−y,  b3(x,y)=13!ex−y,b4(x,y)=14!ex−y,…c0(x,y)=e−x+y,  c1(x,y)=e−x+y,c2(x,y)=12!e−x+y,  c3(x,y)=13!e−x+y,c4(x,y)=14!e−x+y,….

Therefore, we obtain the solutions of (38) as

(48) u(x,y,t)=ex+y−ex+ytαΓ(α+1)+ex+yΓ(α+1)t2αΓ(2α+1) −12!ex+yΓ(2α+1)t3αΓ(3α+1) +13!ex+yΓ(3α+1)t4αΓ(4α+1)−⋯=ex+y(1+∑n=1∞(−1)nΓ((n−1)α+1)tnα(n−1)!Γ(nα+1)),v(x,y,t)=ex−y+ex−ytαΓ(α+1) +ex−yΓ(α+1)t2αΓ(2α+1)+12!ex−yΓ(2α+1)t3αΓ(3α+1) +13!ex−yΓ(3α+1)t4αΓ(4α+1)+⋯=ex−y(1+∑n=1∞Γ((n−1)α+1)tnα(n−1)!Γ(nα+1)),w(x,y,t)=e−x+y+e−x+ytαΓ(α+1) +e−x+yΓ(α+1)t2αΓ(2α+1)+12!e−x+yΓ(2α+1)t3αΓ(3α+1) +13!e−x+yΓ(3α+1)t4αΓ(4α+1)+⋯=e−x+y(1+∑n=1∞Γ((n−1)α+1)tnα(n−1)!Γ(nα+1)).

If we solve (38) with *α* = 1, we obtain the exact solution

(49) u(x,y,t)=ex+y(1−t+t22!−t33!+⋯)=ex+y−t,v(x,y,t)=ex+y(1+t+t22!+t33!+⋯)=ex−y+t,w(x,y,t)=e−x+y(1+t+t22!+t33!+⋯)=e−x+y+t.

## 5. Conclusion

In the present work, we proposed a new homotopy perturbation method to solve nonlinear time-fractional biological population model and a system of three nonlinear time-fractional partial differential equations. The new method for solving fractional-order differential equations is based on two component procedures and polynomial initial condition. The new homotopy perturbation method is very simple in application and is less computational and more accurate in comparison with other mentioned methods. Unlike the Adomian decomposition method, the new homotopy perturbation method is free from the need to use Adomian polynomials. In this method, we do not need the Lagrange multiplier, correction functional, stationary conditions, and calculating integrals, which eliminate the complications that exist in the variational iteration method. The obtained results show that these approaches can solve the problem effectively and can be applied to many nonlinear differential equations. The computations associated with the examples discussed above were performed by MAPLE.
